# Dual analysis of wild-type and attenuated *Orf virus* and host cell transcriptomes revealed novel virus-host cell interactions

**DOI:** 10.1128/msphere.00398-23

**Published:** 2023-11-20

**Authors:** Xiaoting Yao, Tian Jing, Qingru Geng, Ming Pang, Xuanduo Zhao, Shaofei Li, Dekun Chen, Wentao Ma

**Affiliations:** 1 College of Veterinary Medicine, Northwest A&F University, Yangling, Shaanxi, China; University of Michigan, Ann Arbor, Michican, USA

**Keywords:** *Orf virus*, dual RNA sequencing, viral transcriptome, virulence determinant, virus-host interactions

## Abstract

The attenuated *Orf virus* (ORFV) strain (aFX0910), derived from continuous passages of wild-type strain FX0910 *in vitro*, displays obviously lower virulence in kids. However, the latent virulence determinants and intracellular mechanisms for the attenuated variant remain unknown. In this study, we constructed a comprehensive analysis of the ORFV and goat lip fibroblasts transcriptomes during viral infection using RNA sequencing. Focusing on the host transcriptome, we performed gene expression analysis, Gene Ontology analysis, and biological pathway analysis, among others. This integrated analysis allowed us to assess potential changes in mRNA expression and single nucleotide polymorphisms (SNPs) in both viral and host genes. In general, the global patterns of immune gene expression induced by different ORFV isolates were mostly shared. However, host transcripts and non-synonymous of viral genes differed between the wild-type and attenuated ORFV strains. Notably, the natural ORFV isolate (FX0910) predominantly triggered an innate immune response in host cells, while aFX0910 downregulated innate immune response and antiviral response. Analysis of viral transcriptomes manifested that FX0910 and aFX0910 had different SNP events in multiple functional genes. Collectively, our findings highlighted novel insights into virus-host interactions and shed light on the molecular basis of ORFV virulence at the genomic level. Furthermore, this study may contribute to the development of targeted intervention strategies and the advancement of vaccine research in the field of ORFV.

**IMPORTANCE:**

Currently, the only available commercial vaccines for *Orf virus* (ORFV) are live attenuated vaccines, which present a potential risk of reversion to virulence. Therefore, understanding the pathogenic mechanisms of different virulent strains of ORFV and host immune responses triggered by these viruses is crucial for developing new vaccines and interventions. In this study, we found that the attenuated strain downregulates the host innate immune response and antiviral activity. In addition, we noted that the wild-type strain can induce the immune response pattern centered on interferon-stimulated genes and interferon regulatory factor gene family. We predicted that STAT1 and STAT2 are the main transcription factors upstream of target gene promoters through gene regulatory networks and exert significant regulatory effects on co-expressed genes. Our study elucidated the complex interaction between ORFV strains and host cell immune responses, providing new insights into vaccine research for ORFV.

## INTRODUCTION

*Orf virus* (ORFV), a member of the *Parapoxvirus* genus in the poxviridae family, is responsible for contagious ecthyma affecting mostly goats and sheep, posing a great economic threat to breeding industry (1). In addition, increasing evidence has revealed that humans can be infected by contact with diseased animals (2–4). In a whole flock, the infection diffuses rapidly by direct or indirect contact causing up to 90% being affected; the mortality rate is 10%~93% in kids (5, 6). Consequently, there is an urgent need to develop effective vaccines and novel antiviral intervention strategies to mitigate the pathogenicity of ORFV, as well as to overcome drug resistance associated with current antiviral measures (7, 8).

Similar to other poxviruses, ORFV contains a linear double-stranded DNA genome divided into 132 gene segments. The central region of ORFV genome harbors essential genes involved in viral replication and the formation of mature virions. In contrast, the terminal genes, which constitute approximately 20% of the ORFV genome, encode factors associated with virulence, pathogenesis, host range, and immune evasion (9). Inverted terminal repeats (ITR) are found at the genomic terminus and covalently closed with 100-bp hairpin loops. ORFV strains are characterized by extended heterogeneity and exhibit various restriction fragment profiles (10). Comparative analyses of three published ORFV isolates have suggested a substantial level of genetic variation among different species (11).

There are still several challenges to our acquaintance of ORFV pathogenesis and the development of an effective vaccine, particularly in comprehending the role of viral genes in disease causation and their interaction with host cellular gene products. Despite the publication of several ORFV genome sequences in recent years, fundamental questions persist regarding the nature and quantity of viral genes (9, 11, 12).

To address these challenges, we employed dual RNA sequencing (RNA-seq), which provides analyses of host-pathogen transcriptome interactions that can dynamically elucidate their interactions and provide a means to explore the underlying molecular mechanisms (13). This approach provides valuable insight into the responses of both pathogens and host cells during infection (14–16). In our study, we refer to the ORFV natural strain cultured *in vitro* as the wild-type strain, and the attenuated strain is the wild-type strain obtained by continuous passage *in vitro*. We applied dual RNA-seq to investigate the intracellular dynamics of ORFV, specifically examining the gene expression changes occurring in goat lip fibroblasts (GLF) infected with wild-type or attenuated ORFV strains. By comparing the transcriptomes of infected cells between different virulence strains, we identified distinct host responses associated with each strain. Additionally, by integrating measurements of single nucleotide polymorphisms (SNPs), we provide evidence suggesting that missense mutations in viral genes may alter the protein structure, consequently influencing the outcome of ORFV infection in GLF. These findings underscore the significance of employing dual RNA-seq to gain deeper insights into the RNA biology of host cells during ORFV infection.

Overall, our study highlights the value of utilizing dual RNA-seq to elucidate the intricate interactions between ORFV and host cells at the genomic level. By integrating comprehensive transcriptomic analysis and SNP measurements, we enhance our understanding of the biology of host cells infected by ORFV, contributing to the advancement of ORFV research and the development of effective control strategies.

## MATERIALS AND METHODS

### Cell culture, viruses, and infection

GLF were isolated from the lip of goat embryos. The adherent cells were obtained by the same method as previously reported (17) and were treated with trypsin-EDTA (Thermo Fisher Scientific) for 1 min. When most fibroblasts were detached, they were collected and centrifuged at 800 × *g* for 5 min. The supernatant was discarded, and fibroblasts were cultured in Dulbeco’s modified Eagle’s medium (DMEM)/F-12 medium (Thermo Fisher Scientific) supplemented with 10% fetal bovine serum (ExCell Bio) at 37℃ and 5% CO_2_. The natural strain of ORFV was isolated (18) from blood samples with confirmed ORFV infection in Shaanxi. Subsequently, ORFV amplification and culture were conducted on cells following previously described protocols (18). After 11 and 85 consecutive generations of ORFV natural strain proliferation, the virus suspension was coined as FX0910 or aFX0910, respectively (cytopathic effect was observed at the ninth passage of virus). The TCID_50_ and growth curve of the ORFV were calculated by the Reed-Muench method (19). The FX0910 and aFX0910 were inoculated into the inner mucosa of the lower lip of 1-month-old kids, respectively, and the negative control was inoculated with normal saline (three kids per group). GLF were infected with FX0910 or aFX0910 at a multiplicity of infection (MOI) of 5. Following incubation at 37°C for 1 h, the viral suspension was removed and the cells were further incubated in minimum essential medium (MEM) (Thermo Fisher Scientific) supplemented with 2% fetal bovine serum for additional durations of 3 h, 12 h, 24 h, 36 h, 48 h, 60 h, and 72 h. The control group (0 h) was incubated with an equal amount of maintenance medium. Three replicate samples were collected at each time point. All experiments involving animals were performed in accordance with the guidelines of the Care and Use of Laboratory Animals of the Ministry of Health of China. Animal studies of this article were approved by the Research Ethics Committee of Northwest A&F University.

### RNA extraction, library construction, and sequencing

Considering that the majority of cells died at 48 h after FX0910 infection, total RNA was extracted from GLF cultured with FX0910 and aFX0910 at 0 h, 3 h, 12 h, 24 h, and 48 h using RNAiso Plus (TaKaRa). The quality, concentration and RNA integrity number (RIN) of total RNA were analyzed by Agilent 5400 and NanoDrop (RIN ≥ 7.5, 28S/18S ≥ 1.0). The mRNA fragment was used as a template, and random oligonucleotides served as primers to synthesize the first strand of cDNA by a M-MuLV Reverse Transcription System. The RNA strand was then degraded using RNaseH. Simultaneously, the second strand of cDNA was synthesized using the DNA polymerase I system with dNTPs as raw materials. Double-stranded cDNA was purified, followed by the addition of poly-A to the ends and ligation of sequencing adapters (NEBNext Ultra RNA Library Prep Kit for Illumina. Then, cDNA in the size of 250–300 bp were screened by AMPure XP beads. Purification and enrichment of the selected cDNA fragments were performed using PCR to create the final cDNA library. To ensure library quality, the library was diluted to 1.5 ng/µL for preliminary quantitative analysis with a Qubit 2.0 Fluorometer. The insert size was measured with an Agilent 2100 Bioanalyzer, and the effective concentration of the library (>2 nM) was accurately determined by real-time PCR.

### Mapping, quantitative, and PCA analyses of transcriptome data

The raw RNA-seq reads were processed using SOAPnuke 2.1.5 to trim adapters, undetermined bases, and low-quality base reads. Then, the sequence quality was verified using FastQC. Clean reads were mapped to the *Capra hircus* (goat) reference genome (RefSeq assembly accession: GCF_001704415.2) and ORFV reference genome (RefSeq assembly accession: NC_005336.1) using HISAT2. The expression levels of all transcripts were evaluated using the featureCounts package of Subread, and the results were reported as fragments per kilobase per million reads (FPKM) (20). ORFV gene expression was analyzed by unsupervised hierarchical clustering of FPKM with the heatmap package. Principal component analysis (PCA) was performed using the prcomp function and ggplot2 packages.

### Single nucleotide polymorphism analysis

Due to the lack of complete genetic sequences of the strains studied in this work, we compared the reads of FX0910 and aFX0910 with reference genomes, respectively. The maftools package (21), along with the plotmafSummary function, was employed for statistical analysis and visualization of the Mutation Annotation Format (MAF) file containing mutation annotation information of virus samples. The top 10 viral genes with the largest numbers of single nucleotide polymorphisms were selected and compared with the ORFV reference genome to identify the mutated amino acid sites. Homology modeling analysis was conducted using the Phyre2 program (http://www.sbg.bio.ic.ac.uk/phyre2/html/page.cgi?id=index), with a model confidence level of at least 90%.

### Sample clustering and correlation analysis

First, the expression data of the samples were standardized using the scale function, and the Manhattan distance between different samples was calculated by the dist function. The resulting distance matrix was used to generate a sample cluster tree using the plot function. In addition, the core function was employed to analyze the sample correlation, followed by a heatmap of Spearman correlation coefficients in the correlation analysis using the pheatmap package. Volcano diagram was created using the TCGAbiolinks package in R (22).

### Host RNA-seq differential expression and enrichment analysis

The jvenn (https://jvenn.toulouse.inrae.fr/app/example.html) was performed to generate the Venn diagram. DefinedRegionDifferentialSeq normalized the raw data and calculated negative binomial *P* values using DESeq2 (23), followed by false discovery rates (FDR) (23). The *P* value in the hypothesis test was corrected by the *P*_adj_ value, and the differentially expressed gene (DEGs) was *P*_adj_ ≤ 0.05 and |log2(fold change)| ≥ 1. Gene ontology (GO) (24) and Kyoto Encyclopedia of Genes and Genomes (KEGG) (25) analyses were used to determine significantly enriched GO terms and pathways (*P* ≤ 0.05). Subsequently, gene set enrichment analysis (GSEA) of GO and KEGG gene sets in goats was conducted by fgsea package (26).

### Weighted correlation network analysis and transcription factor prediction

A weighted gene co-expression network was constructed by the weighted correlation network analysis (WGCNA) package (27). DEGs were clustered and outliers were eliminated. The selection of the soft threshold was performed using the pickSoftThreshold function, which is based on the scale-free topology criterion; then, the topological overlap matrix (TOM) was constructed. Module characteristic genes were identified, and their association with sample phenotypic traits was assessed based on correlation coefficients and *P* values. The resulting network map was visualized using STRING and Cytoscape, highlighting important network modules and key related genes. To identify significantly enriched metabolic pathways and their upstream regulatory factors, the iRegulon plugin in Cytoscape was utilized to analyze co-expressed genes in samples. Predicted regulators and targets were selected within a 500-kb upstream region, with a normalized enrichment score (NES) value > 3.5, FDR < 0.01.

### Quantitative real-time PCR and statistical analysis

Real-time PCR was employed to determine the expression of STAT1, STAT2, OAS1, IFI6, CXCL5, CXCL8, CXCL10, ORFV016, ORFV017, ORFV020, and ORFV125. Total RNA (1 µg) was reverse transcribed with a Monad first-strand cDNA synthesis kit (5× RTIII MonScriptTM All-in-One, Mix with dsnase). Then, cDNA samples were amplified using MonAmp ChemoHS qPCR Mix (Monad) on a Roche LightCycler 480 Real-Time PCR System. The reaction conditions consisted of 1 µL of cDNA, 0.4 µL of 10 µM primers, and 10 µL qPCR Mix in a final volume of 20 µL. Predeformation at 95°C for 10 min, each cycle consisted of denaturation at 95°C for 10 s, annealing, and extending at 60°C for 30 s, for a total of 40 cycles. β-Actin was used as the reference gene, and the primer sequences used for each gene were as follows: β-actin, forward (5′-CCTGCGGCATTCACGAAAC-3′) and reverse (5′-GGGGGCGCGATGATCTTGA-3′); STAT1, forward (5′-GATCTCTAACGTCTGTCAGCTG-3′) and reverse (5′-GAGGTCCAGGATTCCTTCGATC-3′); STAT2, forward (5′-CCCCCATGGCGCAGTGGGAAATGCTG-3′) and reverse (5′-GGGGAATTCCTAGAAGTCAGAAGGCATC-3′); OAS1, forward (5′-TCATCCGCCTGGTGAAGCACTGG-3′) and reverse (5′-CTGGGGAAAGAACTCTCCGA-3′); IFI6, forward (5′-TGCTCTCCTCCAAGATACGG-3′) and reverse (5′-CGCAGGTGTAGAGTAGCAGG-3′); CXCL5, forward (5′-CTCAAGCTGCTCCTTTCTCG-3′) and reverse (5′-GCGATCATTTTGGGGTTAAT-3′); CXCL8, forward (5′-CCCCCATGGTTCAGAAGATTG-3′) and reverse (5′-TTGTCAGAAGCCAGCGTTCAC-3′); CXCL10, forward (5′-CTGTCGTTCTCTGCCTCGTG-3′) and reverse (5′-GGATCCCTTGAGTCCCACTCA-3′); ORFV016, forward (5′-AACGGCACCATGTATCGGTC-3′) and reverse (5′-CTGGACGCGCTCTACTTCG-3′); ORFV017, forward (5′-CCTGCGTGCTCAAGATGGAC-3′) and reverse (5′-TGCTCGCCGTTGTTTTTGG-3′); ORFV020, forward (5′-CTCGGTGTCGGAGTTTTCGT-3′) and reverse (5′-CATGCTGGAATTGGACGACG-3′); ORFV125, forward (5′-CCAACCCTGAAAACTCGCAC-3′) and reverse (5′-GTACACGAGCCGCATGAAGT-3′); ORFV017, forward (5′-CCTGCGTGCTCAAGATGGAC-3′) and reverse (5′-TGCTCGCCGTTGTTTTTGG-3′).

The relative expression levels of the target genes, normalized to β-actin, were calculated by the 2^−∆∆Ct^ method. All experiments were performed in triplicates. Student’s *t*-test was used for statistical significance analysis, and *P* ≤ 0.05 was statistically significant.

## RESULTS

### RNA-seq of ORFV

**Fig 1:**
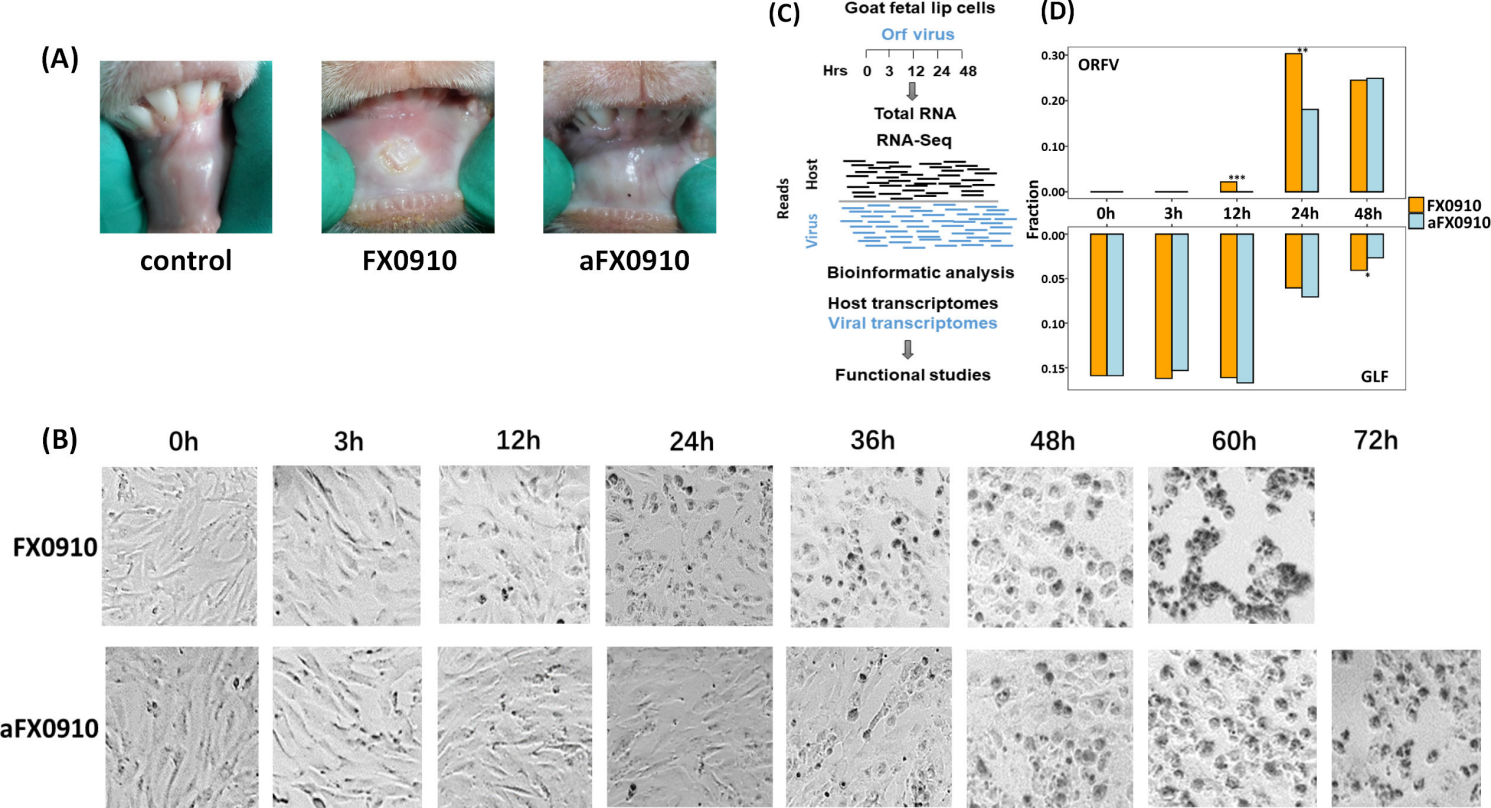
Pathological changes after infection with FX0910 and aFX0910 and dual RNA-seq. (**A**) Pathological changes of lip in lambs infected with FX0910 and aFX0910. (**B**) Morphological changes of GLF at different time points after infection with FX0910 and aFX0910. (**C**) The schematic of RNA-seq approach. All RNA samples were extracted from FX0910- and aFX0910-infected cells at time points 0 h, 3 h, 12 h, 24 h, and 48 h and then subjected to an RNA-seq assay that maintains the host and virus RNA. (**D**) RNA-seq read counts were showed by several hours post-infection (hpi) (*x*-axis) as well as their read fraction distribution (*y*-axis).

To assess the virulence phenotype of FX0910 and aFX0910 strains, goats were infected with 100 TCID_50_ of virus by scratch inoculation inside the lower lip and observed for 10 days for signs of ecthyma and crust. Kids infected with FX0910 or aFX0910 exhibited characteristic skin lesions consistent with previous research on ORFV infection (28, 29). The difference was that FX0910 induced more severe symptoms compared with aFX0910 (Fig. 1A).

To investigate the host cell response to ORFV infection, GLF were infected with ORFV (Fig. 1B). Dual RNA-seq was performed by Illumina technology, and all reads were mapped to the reference genome of ORFV and, in parallel, the goat genome. A schematic of our experiment is shown in Fig. 1C. Upon mapping the sequencing data to the reference genomes of goat and ORFV, it was showen that the amount of sample sequencing data is between 37,313,880 reads and 56,812,084 reads. The mapping rate with the goat reference genome is between 90.20% and 93.69% (3 h to 12 h after infection) and 9.92% and 48.46% (24 h to 48 h after infection). However, the mapping rate with the ORFV reference genome was between 0.00%–2.86% (3 h to 12 h after infection) and 25.32%–39.29% (24 h to 48 h after infection) (Table 1; Table S1).

**TABLE 1:** Percentages of read counts that mapped to goat and ORFV reference genomes

Sample	Goat (%)	All reads	ORFV (%)
C1_0h	44,158,861 (93.51%)	47,839,650	0 (0.00%)
C2_0h	40,282,138 (92.44%)	44,314,914	0 (0.00%)
C3_0h	40,694,274 (90.82%)	45,484,584	0 (0.00%)
FX0910_3h_1	44,674,036 (93.3%)	48,371,712	16,521 (0.034%)
FX0910_3h_2	38,994,251 (93.18%)	42,360,524	15,690 (0.037%)
FX0910_3h_3	45,732,222 (93.51%)	49,876,106	17,175 (0.034%)
aFX0910_3h_1	42,513,948 (93.65%)	45,956,486	0 (0.00%)
aFX0910_3h_2	36,707,460 (93.53%)	40,068,250	0 (0.00%)
aFX0910_3h_3	43,192,747 (93.31%)	47,029,230	0 (0.00%)
FX0910_12h_1	42,802,635 (91.24%)	47,476,636	1,234,942 (2.6%)
FX0910_12h_2	41,346,999 (90.2%)	46,706,916	1,335,996 (2.86%)
FX0910_12h_3	44,472,315 (90.54%)	49,925,420	1,291,058 (2.59%)
aFX0910_12h_1	45,423,725 (93.55%)	50,911,732	0 (0.00%)
aFX0910_12h_2	43,007,722 (93.58%)	46,505,152	0 (0.00%)
aFX0910_12h_3	45,272,014 (93.69%)	49,029,812	0 (0.00%)
FX0910_24h_1	13,290,207 (30.73%)	43,248,770	16,153,157 (37.35%)
FX0910_24h_2	11,969,515 (27.46%)	43,588,412	17,123,824 (39.29%)
FX0910_24h_3	18,898,019 (33.26%)	56,812,084	20,709,720 (36.45%)
aFX0910_24h_1	18,290,970 (47.23%)	38,726,772	10,414,194 (26.89%)
aFX0910_24h_2	15,053,776 (40.34%)	37,313,880	10,784,563 (28.9%)
aFX0910_24h_3	21,023,281 (48.46%)	43,381,036	10,982,552 (25.32%)
FX0910_48h_1	10,918,098 (25.57%)	42,694,952	13,889,117 (32.53%)
FX0910_48h_2	9,131,498 (21.9%)	41,693,584	15,251,722 (36.58%)
FX0910_48h_3	7,997,414 (20.43%)	39,147,120	14,477,930 (36.98%)
aFX0910_48h_1	4,350,329 (9.92%)	43,860,602	16,871,145 (38.47%)
aFX0910_48h_2	4,929,520 (11.83%)	41,673,934	14,019,846 (33.64%)
aFX0910_48h_3	4,684,099 (11.81%)	39,650,026	13,366,919 (33.71%)

### Viral genomics of FX0910 and aFX0910 ORFV strains

**Fig 2:**
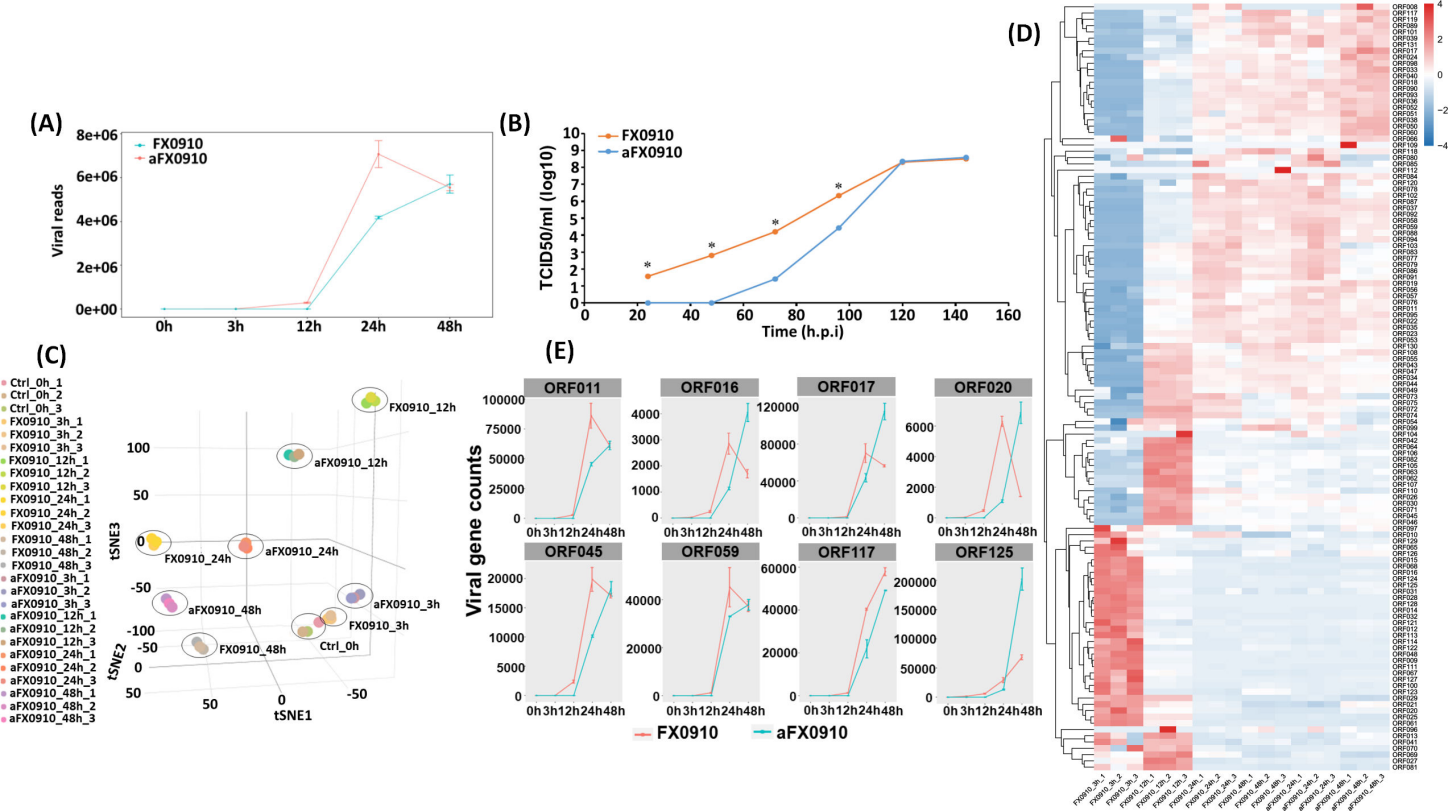
ORFV genomics and gene clustering. The viral growth curves were obtained by normalizing viral reads for FX0910 (orange) and aFX0910 (blue) (**A**) and confirmed by viral TCID_50_ in GLF (**B**). (**C**) Principal component analysis suggesting biological replicates for all samples. (**D**) Hierarchical clustering of FX0910 and aFX0910 genes at different infection times. (**E**) Each viral segment count (gray boxes) was showen in this plot to reveal the temporal expression of ORFV RNAs.

The distribution of ORFV and host read counts for all samples were shown in Fig. 1D. After normalizing ORFV and GLF read coverage, the RNA-based growth curve of FX0910 and aFX0910 was generated (Fig. 2A). Besides, the growth curve of these two viruses was also confirmed by viral titration in GLF (Fig. 2B). PCA revealed that all 3 hpi clustered with 0 hpi controls; however, FX0910 and aFX0910 at other time points were relatively discrete (Fig. 2C). The expression of virus genes at different times showed that the major gene groups expressed by ORFV changed with different stages of infection (Fig. 2D). It provides reference for studying the function of the virus gene.

Considering that aFX0910 did not exhibit detectable viral gene transcripts at 3 and 12 hpi in host cells, the subsequent analysis focused on the viral transcriptome data at 24 and 48 hpi. The differential expression of viral genes between FX0910 and aFX0910 was compared (Fig. S1), showing that the expression of ORF016, ORF017, ORF020, and ORF125 genes in aFX0910 was significantly higher than that in FX0910 at 48 hpi. The expression of these genes was evaluated by real-time PCR (Fig. S2). The relative temporal expression of viral gene segments was tracked by analyzing the statistics of viral gene counts (Fig. 2E), which exhibited consistency with the growth curves of FX0910 and aFX0910 (Fig. 2A). Prior to 24 hpi, all gene segments of FX0910 displayed higher expression abundance than those of aFX0910, thereby confirming the growth kinetics of these two ORFV strains as depicted in Fig. 1B.

### FX0910 and aFX0910 different gene mutations

**Fig 3:**
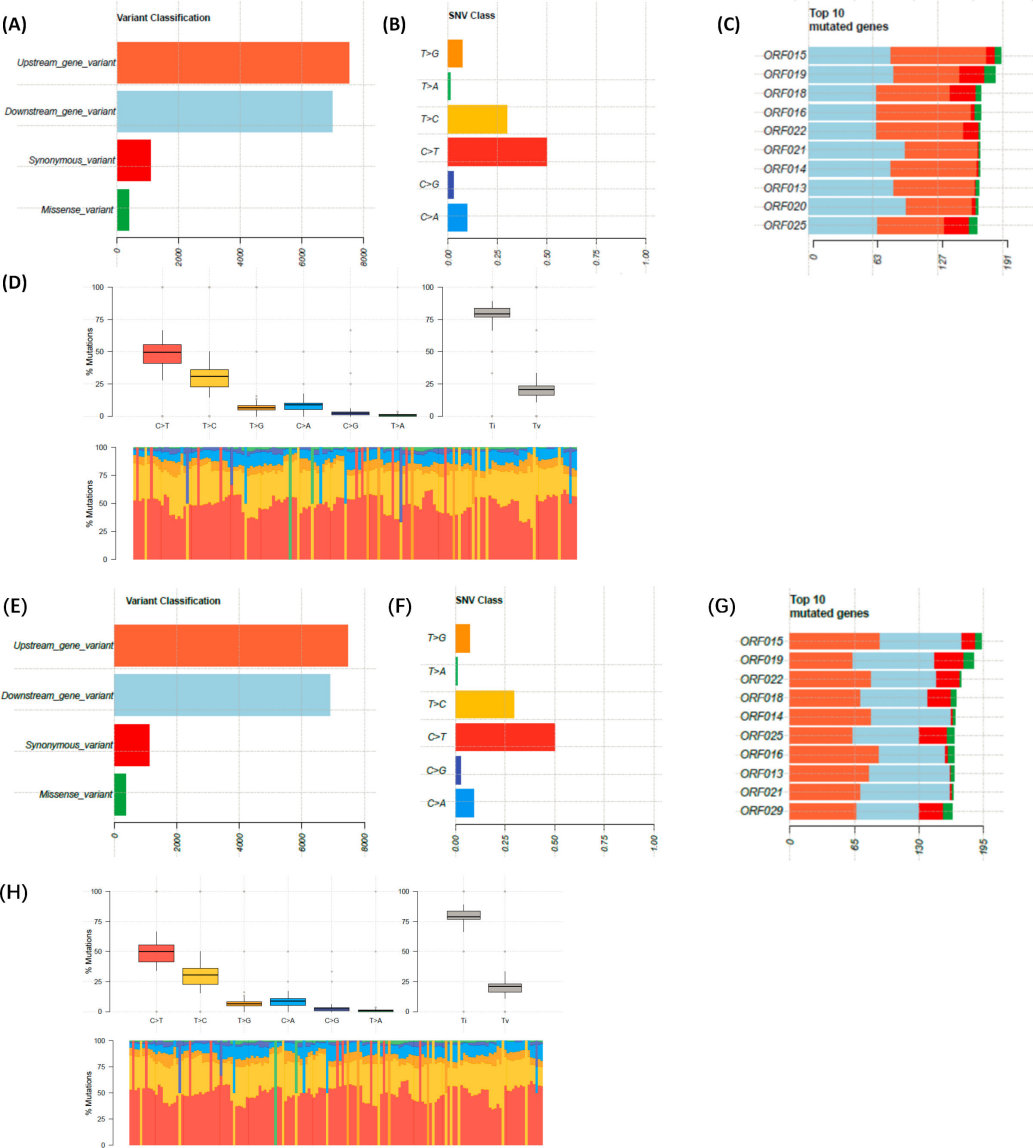
Analyze and visualize MAF files of FX0910 and aFX0910, where panels **A to D** are the results obtained by FX0910 and panels **E to H** are the results obtained by aFX0910. (**A, E**) Mutation classification statistics; (**B, F**) detailed statistics of SNP mutations; (**C, G**) top 10 genes with the most mutations; (**D, H**) summary graph of transitions and transversions, the upper left plot shows the base statistics of mutation. The upper right plot shows the ratio of base transversion and conversion, Ti represents conversion, Tv represents transversion, the lower plot is a stacked bar chart of the proportion of base mutations in all samples, the color corresponds to the boxplot, and the overall percentage is similar to the boxplot.

In the process of virus infection in host cells, the determination of viral gene mRNA can provide more insight into the new elements of host-virus interaction during virus infection in host cells. Next, we performed SNP analysis of FX0910 and aFX0910 strains to determine whether virulence affects the viral gene variation (Fig. 3). Compared with aFX0910, the mutation classification was similar with FX0910, that is, the occurrence of synonymous mutations is significantly higher than that of missense mutations (*P* < 0.05) (Fig. 3A and E). The distribution of single nucleotide variant showed the most frequent conversion of C>T (Fig. 3B, D, F, and H). In addition, the top 10 mutation genes showed a high degree of similarity between FX0910 and aFX0910 (Fig. 3C and G), indicating a consistent mutation profile between the two strains.

**Fig 4:**
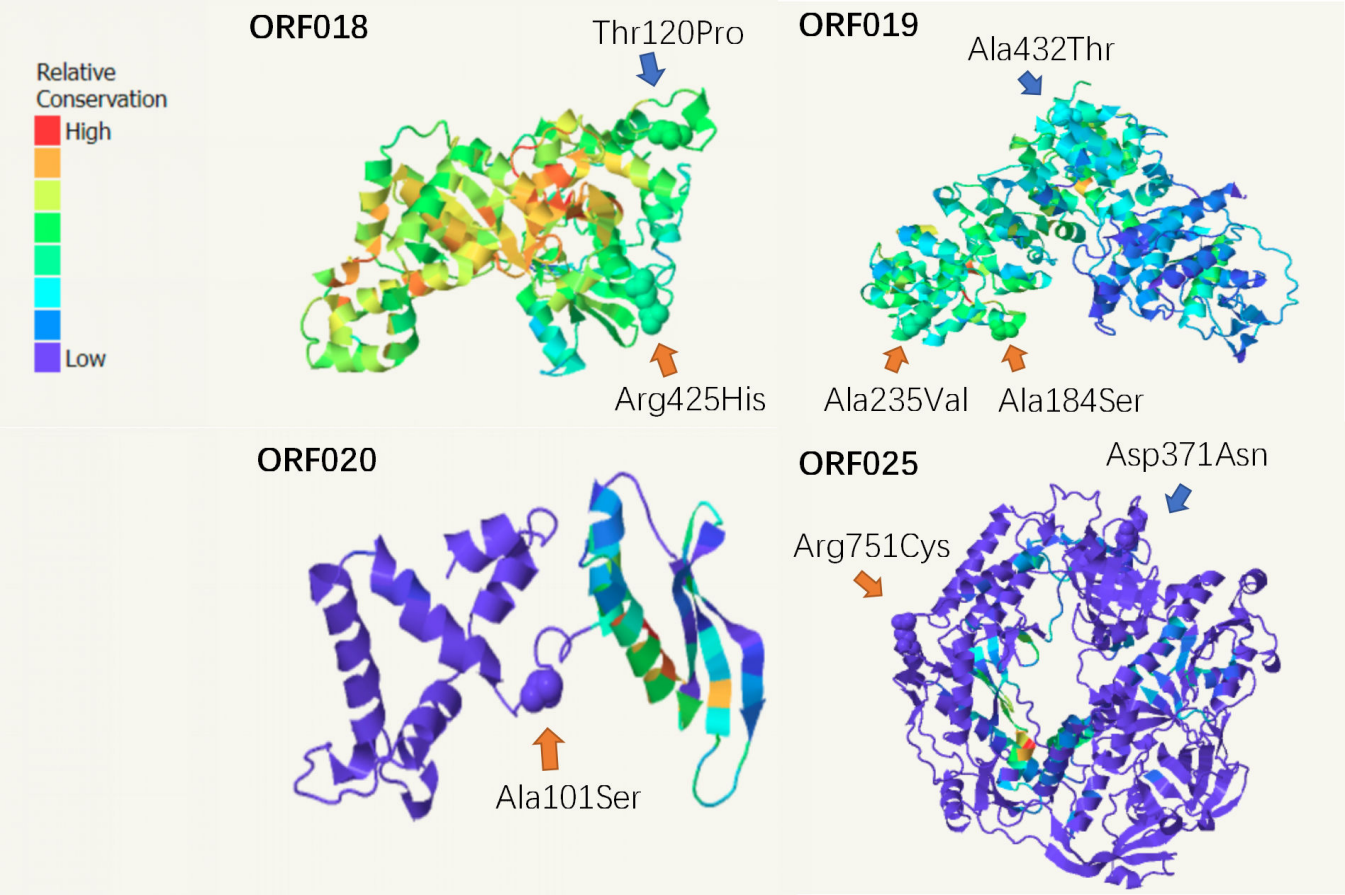
Homology modeling of ORFV. (**A**) ORF018; (**B**) ORF019; (**C**) ORF020; (**D**) ORF025. The yellow arrow in the plot indicates the mutation site of the FX0910 strain, and the blue arrow indicates the aFX0910 mutation site.

Based on the SNP information, we further detected whether it influenced the tertiary structure of proteins. The results of homology modeling analysis obtained proteins (ORF018, ORF019, ORF020, and ORF025) (Fig. 4; Table 2). In the FX0910 strain, a variation at amino acid position 425 (Arg to His) was observed in ORF018, while in the aFX0910 strain, a variation at position 120 (Thr to Pro) was detected. For ORF019, two mutation sites were identified in the FX0910 strain, at positions 184 and 235 (Ala to Ser, Ala to Val), while in the aFX0910 strain, the mutation occurred at position 432 (Ala to Thr). In the FX0910 strain, a mutation was found at position 101 (Ala to Ser) in ORF020, whereas no mutation in the amino acid site was observed in the aFX0910 strain. Finally, in ORF025, a mutation at position 751 (Arg to Cys) was detected in the FX0910 strain, while in the aFX0910 strain, a mutation at position 371 (Asp to Asn) was observed.

**TABLE 2:** ORF018, ORF019, ORF020, and ORF025 amino acid variation sites

Gene	FX0910		aFX0910	
	Position	Variation	Position	Variation
ORF018	425	Arg to His	120	Thr to Pro
ORF019	184	Ala to Ser	432	Ala to Thr
	235	Ala to Val		
ORF020	101	Ala to Ser	–^*a*^	–^*a*^
ORF025	751	Arg to Cys	371	Asp to Asn

^*a*^ “–” indicates no contents.

### aFX0910 infection downregulated the innate immune response and antiviral activities

**Fig 5:**
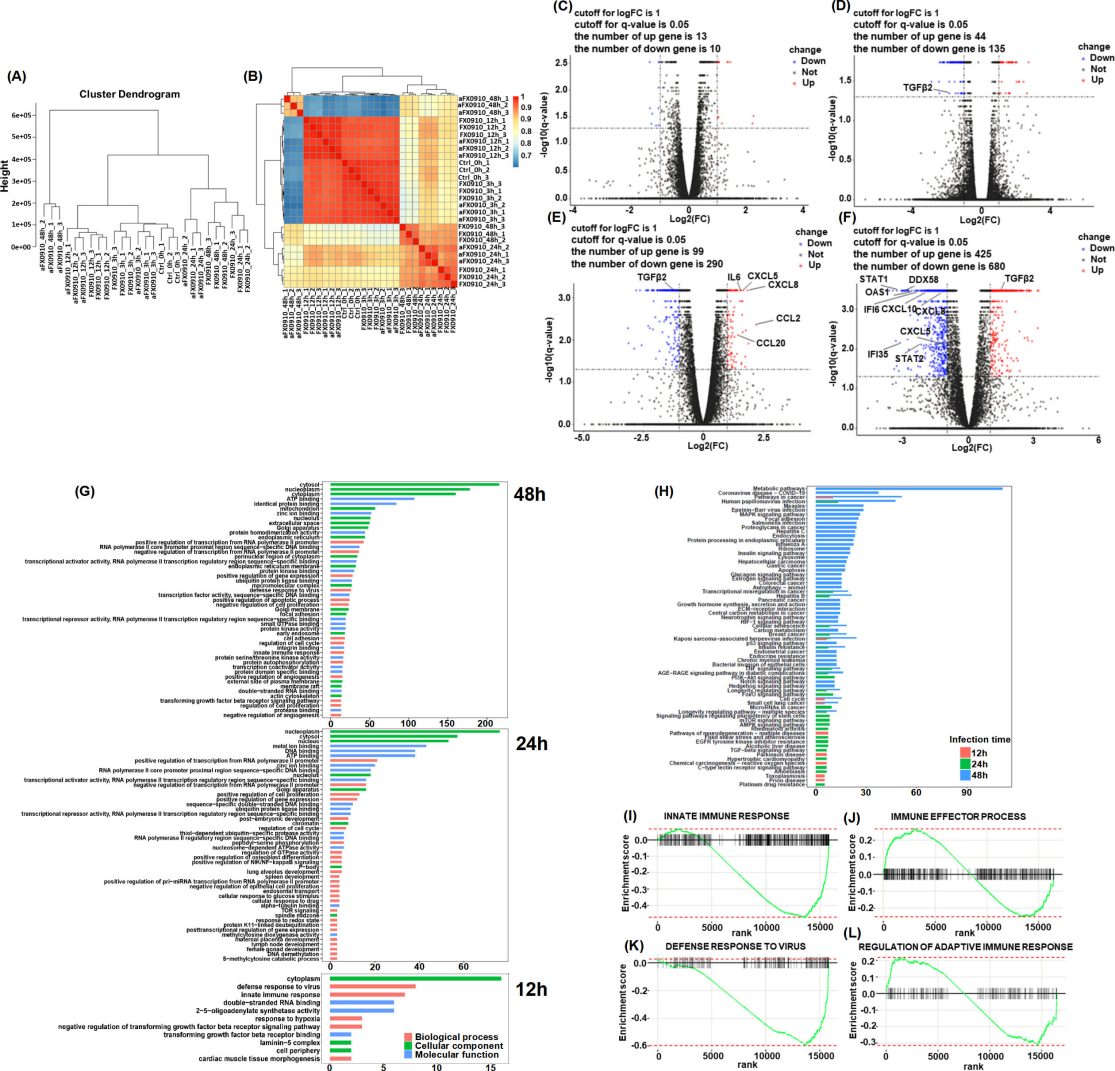
Differentially expressed genes in ORFV-infected samples. (**A, B**) Cluster analysis between different cell samples. The clustering relationship between different samples is displayed by (**A**) Manhattan distance and (**B**) Spearman correlation coefficient, respectively. (**C–F**) Volcano plot of DEGs in host cells. Differentially expressed genes at 3 h (**C**), 12 h (**D**), 24 h (**E**), and 48 h (**F**) after aFX0910 infection compared with FX0910 infection; in the plot, red point indicates upregulated genes and blue point indicates downregulated genes. (**G**) GO enrichment analysis of DEGs. The functional annotation results of DEGs in cells infected with FX0910 and aFX0910 at 3 h, 12 h, 24 h, and 48 h were compared. (**H**) KEGG enrichment analysis of DEGs. The signal pathway results of DEGs in cells infected with FX0910 and aFX0910 at 3 h, 12 h, 24 h, and 48 h were compared. (**I–L**) GSEA enrichment analysis of DEGs. The enrichment of DEGs in the signal pathway of (**I**) innate immune response, (**J**) immune effector process, (**K**) defense response to virus, and (**L**) regulation of adaptive immune response.

In order to identify the differences in gene expression patterns of samples at diverse times of infection, we performed cluster analyses on the transcript data of all samples. The samples were successfully grouped together (Fig. 5A), indicating their similar infection stages. In addition, the intragroup Spearman correlation coefficient for all cell samples exceeded 0.9 (Fig. 5B), demonstrating high reproducibility within the sample group. Notably, intergroup correlations revealed distinct cell gene expression patterns at different time points after infection with different virulent strains. Besides, chemokines related to immune cells exhibited differential expression in host cells at 24 to 48 hpi between FX0910- and aFX0910-infected cells (Fig. 5C through F), including IL-6, CXCL5, CXCL8, CCL2, and TGF-β2.

To elucidate the impact of DEGs in host cells after infection with different virulence strains, GO analysis was performed based on these differential genes (Fig. 5G). The analysis revealed enrichment of biological processes associated with “positive regulation of transcription,” “defense response to virus,” and “"positive regulation of gene expression,” “positive regulation of gene expression,” “negative regulation of cell proliferation,” “cell adhesion,” and “innate immune response,” indicating that different virulence strains caused different host responses after infecting host cells, thereby activating different biological regulation and metabolic pathways. Identified enriched cellular component terms associated with “cytosol,” “nucleoplasm,” “cytoplasm,” “nucleolus,” “Golgi apparatus,” and “endoplasmic reticulum,” suggesting different cellular components were involved in the infection and replication of different virulence strains. Enriched molecular functions were defined as associated with “ATP binding,” “zinc ion binding,” “protein homodimerization activity,” and “transcriptional activator activity,” implying that different virulence strains cause different virus and host interactions.

The same with GO analysis, DEGs were significantly enriched in the classifications of the “MAPK signaling pathway,” “Apoptosis,” and “Cellular senescence” in KEGG analysis (Fig. 5H), showing that ORFV with different virulence could induce different intensities of host response. The classification of the “Notch signaling pathway,” “FoxO signaling pathway,” “mTOR signaling pathway,” and “TGF-β signaling pathway” was also enriched, indicating that different virulence ORFV-infected host cells caused a different degree of activation of signaling pathways. In order to more comprehensively investigate the enrichment of DEGs in metabolic pathways of interest, we performed GSEA analysis of DEGs for metabolic pathways related to antiviral infection (Fig. 5I through L). Compared with the FX0910 infection group, the expression levels of genes involved in innate immune response and antiviral activities were significantly downregulated after aFX0910 infection, while certain genes involved in immune effectors and immune regulation displayed significant upregulation.

### Expression of immune response-associated genes post-FX0910 and aFX0910 infections

**Fig 6:**
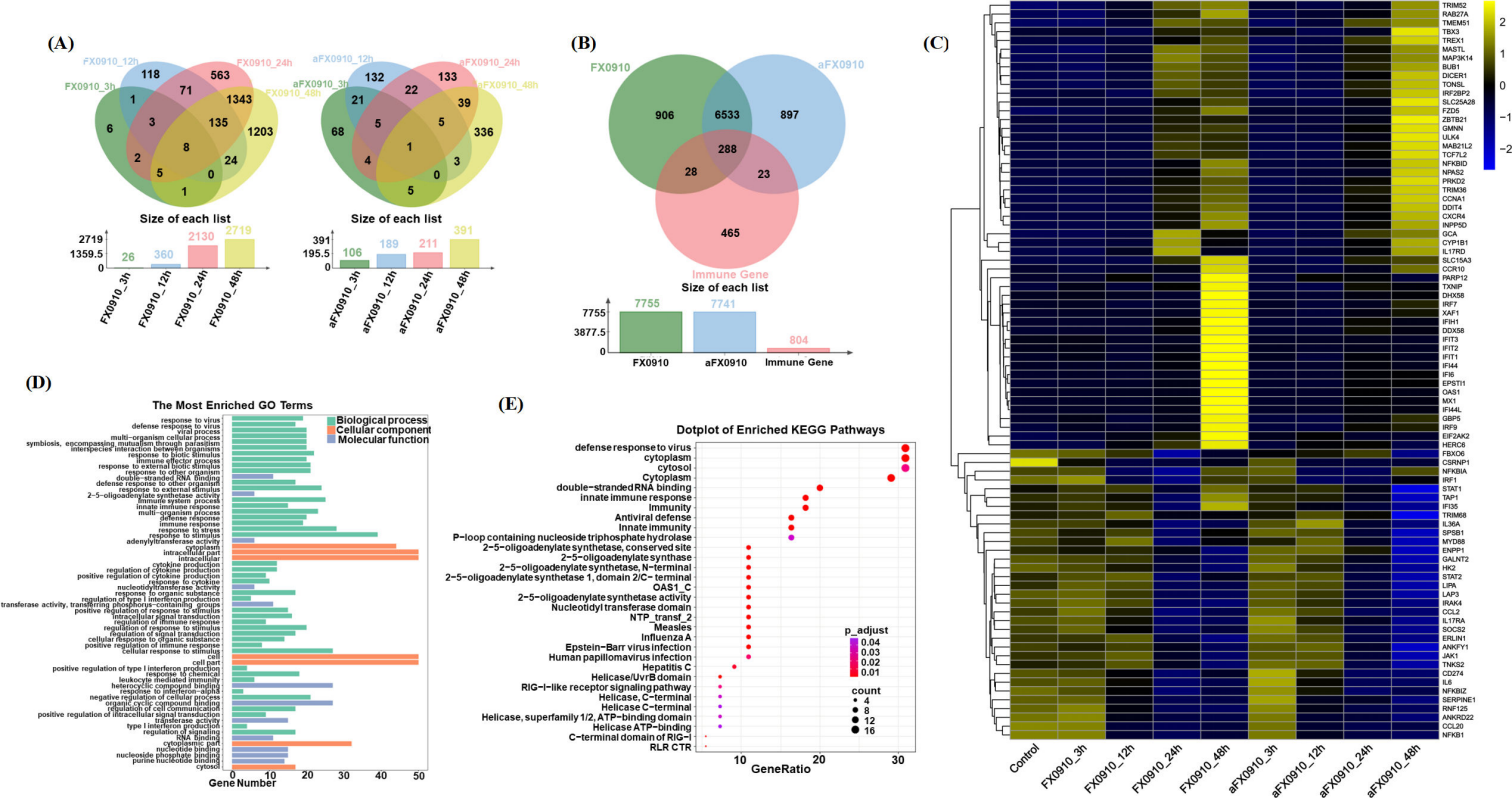
Expression of immune response-associated genes post-FX0910 and aFX0910 infections. (**A**) Expression profile of host genes at different infection times. Left plot shows the differentially expressed gene data set at different time points after FX0910 infection; right plot shows the DEG data set at different time points after aFX0910 infection. (**B**) Venn diagram of “immune-related genes” differentially expressed in response to FX0910 and aFX0910. (**C**) Hierarchical clustering of the 339 DEGs from the “immune-related genes” group. Heat map analysis of host transcriptome showing differentially up- and downregulated genes (yellow and blue, respectively) in FX0910- and aFX0910-infected cells compared with controls. (**D, E**) After FX0910 and aFX0910 infection, GO functional annotation (**D**) and KEGG metabolic pathway enrichment (**E**) were performed with immune-related genes which were significantly upregulated in DEGs induced by host cells.

To date, the immune response of goat cells to different virulence ORFVs has been rarely reported. Therefore, we aimed to investigate the differences in induction of innate immunity pathways at the mRNA level between FX0910 and aFX0910 strains. Following FX0910 and aFX0910 infections, we identified a total of 5, 235, and 897 DEGs (Fig. 6A). Among them, eight DEGs (RF00100, NXPH2, SEC61G, GADD45G, TUBAL3, LIPE, CSRNP1, and FAM20A) were shared across different comparison groups after FX0910 infection. However, only CXCL8 showed significance in aFX0910-infected cells.

Subsequently, we compiled a list of 804 innate immune genes from multiple published references (Table S2) (30–37) and regulatory factors of IFN-β and IFN-related genes (38). Among the 804 innate immune-related genes, 339 were upregulated or downregulated during infection (|log2FC| > 1), while 465 were not differentially expressed (Fig. 6B). Based on these 339 DEGs, 84 DEGs with |log2FC| > 2 were further screened. A heat map was generated (Fig. 6C) to visualize the expression patterns of these 84 DEGs using unsupervised hierarchical clustering analysis. The expression of certain genes was confirmed by real-time PCR (Fig. S3). The upregulated genes were predominantly observed at 48 h post-infection, and distinct DEG patterns were observed in GLF induced by FX0910 and aFX0910 infections. The genes upregulated in GLF infected with FX0910 primarily included antiviral IFN-stimulating genes (IFIT families: IFIT1, IFIT2, and IFIT3), key signaling molecules (IRF9 and IRF7) affecting IFN-I and IFN-III, IFN-induced genes (IFI44 and IFI6), and the IFN-induced enzyme OAS1 with antiviral activity.

To further elucidate the function of the significantly upregulated genes in the above DEGs and the signaling pathways in which they participate, we performed GO and KEGG enrichment analyses based on these genes (Fig. 6D and E). The results showed that its DEGs were mainly present in the cytoplasm and responded to pathogens and external stimuli, including the defense response to viruses, innate immune responses, and RIG-I-like receptor signaling pathways involved in stress responses and post-translational modifications. In addition, the functions of these DEGs include positive regulation of cytokine production, nucleotidyl transferase activity, and positive regulation of IFN-I.

### WGCNA revealed the main immune response pattern centered on ISGs and IRF gene family after FX0910 infection

**Fig 7:**
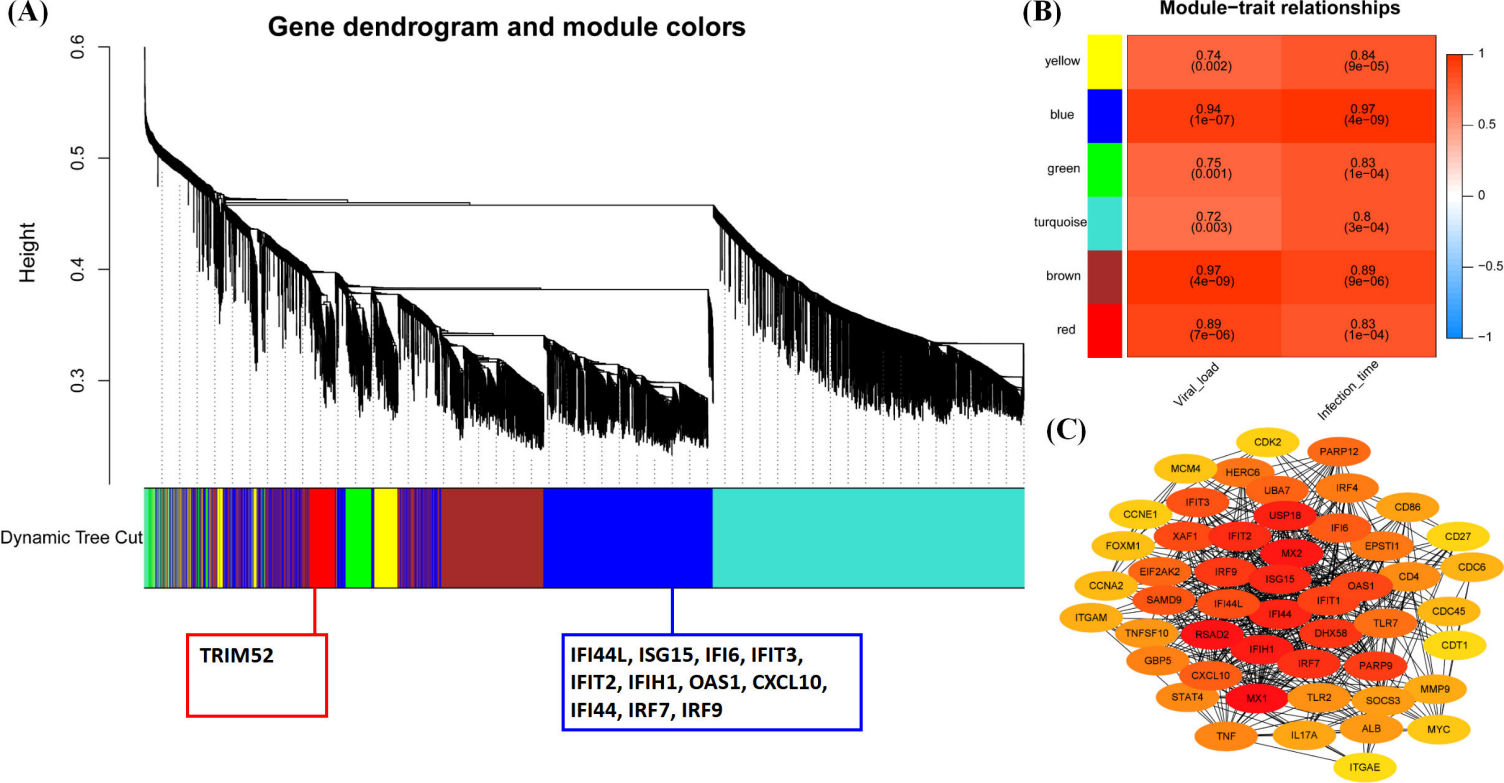
Weighted gene co-expression network and hub gene analysis. (**A**) A hierarchical cluster tree indicating co-expression modules identified with WGCNA. Modules were related to branches and were labeled by colors and showed by the color band. Immune genes were labeled under these color bands. (**B**) Module-trait associations. Every row represented a module eigengene, and column represented a trait. In the plot, each cell included the corresponding correlation and the *P* value. The plot was color coded by correlation based on the color legend. (**C**) Visualization of the connection network among the top 50 connected genes in blue module.

According to the above results, infection of host cells with the FX0910 strain elicits a robust innate immune response. To further investigate this response, we performed WGCNA analysis based on the gene expression profiles at different stages. This analysis aimed to identify hub genes associated with the FX0910 infection. We observed the co-expression of genes in six modules (blue, brown, green, red, turquoise, and yellow) across different stages (Fig. 7A), with the turquoise module containing the largest number of genes. Notably, the blue module primarily encompasses genes involved in the antiviral infection process, exhibiting significant upregulation as depicted in Fig. 6C.

Subsequently, we computed the correlation between gene expression and viral load, as well as infection time, for each module using the corresponding feature vectors (Fig. 7B). The analysis revealed that the blue module exhibited the highest correlation with these two influencing factors, displaying correlation coefficients of 0.94 and 0.97 (*P* < 0.01), respectively. Furthermore, we constructed a gene interaction network for the blue module by importing its gene list into the STRING interaction database. Figure 7C illustrates the top 50 hub genes in the blue module, highlighting intricate interactions among key genes such as IRF9, ISG15, IFI44, IFIT2, IFIT1, OAS1, IFIH1, and IRF7. These findings provide valuable insights into the innate immune response triggered by FX0910 infection and shed light on the critical roles played by hub genes in the blue module.

### STAT2 and STAT1 regulated immune-related co-expression genes during FX0910 infection

**Fig 8:**
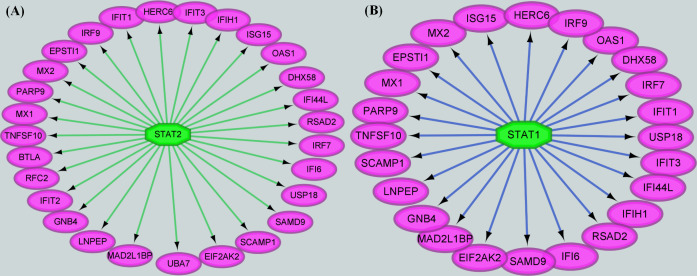
Prediction of upstream regulators of co-expressed genes during FX0910 infection. STAT1 and STAT2 regulate differential expression of co-expressed genes during viral infection.

Transcription factors play a pivotal role in precisely regulating gene expression, influencing various biological processes. In light of the co-expressed genes identified earlier, we constructed a gene regulatory network to predict the transcription factors responsible for regulating these genes following FX0910 infection. Notably, STAT1 and STAT2 emerged as the primary transcription factors upstream of the target gene promoters, exerting significant regulatory control over the co-expressed genes (Table S3; Fig. 8).

**Fig 9:**
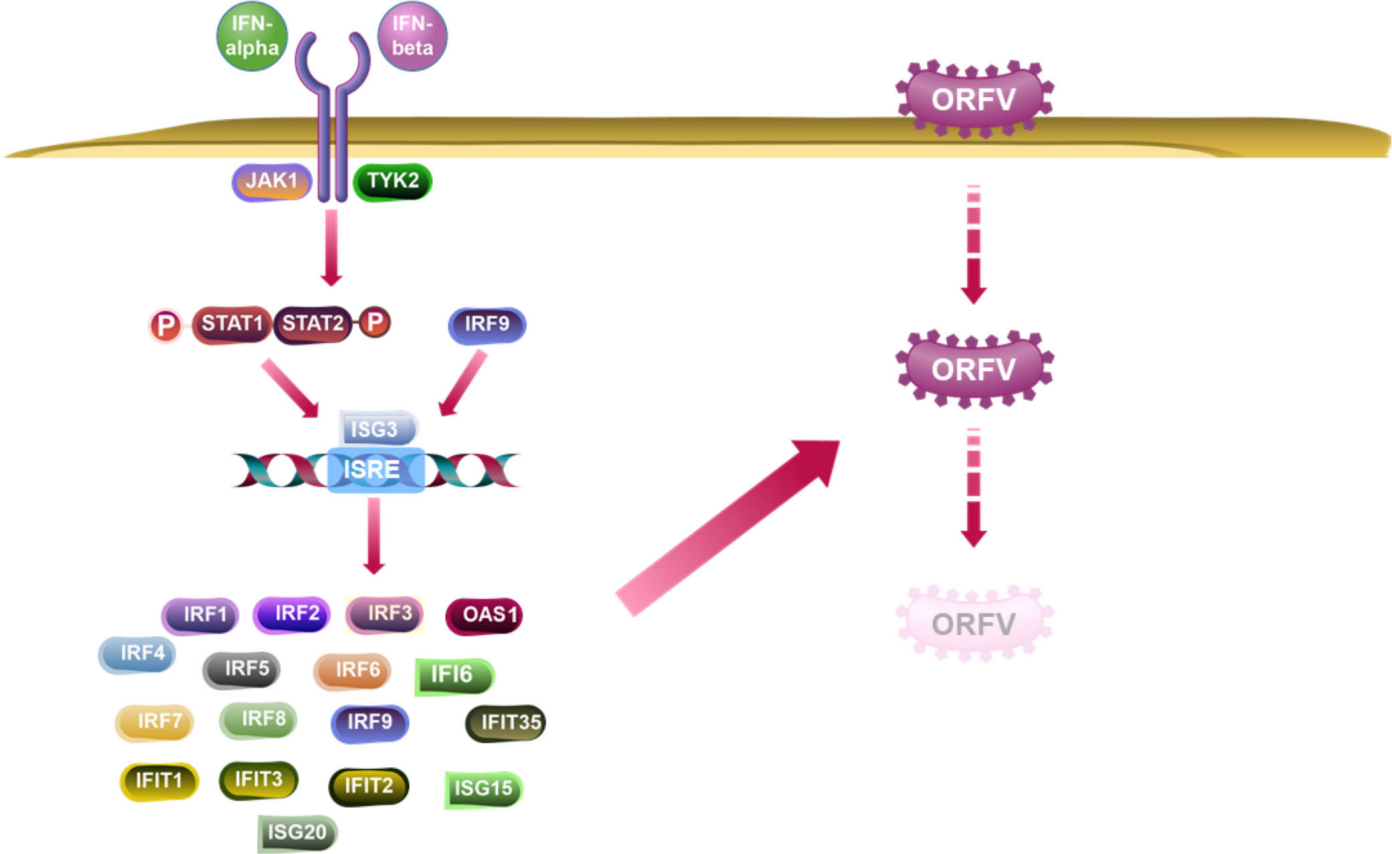
Biological pathways of co-expression genes. Infected GLF with FX0910-induced activation of multiple antiviral genes in the canonical interferon signaling pathway compared with uninfected GLF.

To gain further insight into the functional pathways associated with the co-expressed genes, we explored their involvement in IFN signaling, activation of antiviral genes, and their role in pathogen recognition. The analysis revealed that a cascade of genes induced by STAT proteins directly contributes to the antiviral effects observed during the innate immune response following pathogen infection in host cells (Fig. 9). These findings underscore the critical role of transcription factors, particularly STAT1 and STAT2, in orchestrating the expression of co-expressed genes. Moreover, they shed light on the intricate mechanisms underlying the innate immune response and highlight the significance of IFN-related pathways and antiviral gene activation in pathogen recognition.

## DISCUSSION

Previous studies have analyzed transcriptome data of ORFV-infected host cells and characterized gene expression changes in host cells after ORFV infection (39, 40). However, there are few data on the transcriptome of virus and host and the interaction between virus and host after the infection of host cells with ORFV virulent and attenuated strains by RNA sequencing technology. In this study, we employed dual RNA-seq to simultaneously sequence infected host cells and identify differentially expressed genes of both the host and the virus post-infection. We aim to unravel the underlying intracellular response mechanisms triggered by different virulent ORFV strains upon host cell infection.

Notably, the observed changes in cell phenotype suggest that viral virulence may impact the timing of syncytial production (41). A higher virulence is associated with more rapid virus expansion within host cells, while attenuated strains may gradually adapt to the cellular environment during successive passages. aFX0910 began to proliferate in the middle and late stages, possibly in the early stage to ensure the state and number of cells in order to facilitate its own rapid proliferation in the later stage (11, 42, 43). These findings contribute to a better understanding of the intricate interactions between the host and ORFV strains of varying virulence, providing valuable insights into the cellular and molecular dynamics triggered by viral infection. The results of the study on specific virulence genes show that ORF011, ORF016, ORF017, ORF020, ORF045, ORF059, ORF117, and ORF125 are rapidly upregulated in aFX0910 from 24 h post-infection, and their expression levels exceed those in FX0910. This is consistent with the results in the viral growth curve mentioned above, indicating that aFX0910 is more adapted to the host cell environment during long-term cultivation. Therefore, under the condition of satisfying host cell growth, aFX0910 begins to proliferate rapidly at the late stage of infection.

Different virulence of ORFV produced diverse variations in host cells. Although SNP analysis have been studied in many diseases, limited reports have investigated SNPs in virulent and attenuated ORFV strains and their significance in the context of viral infection (44, 45). The high-frequency mutation of C>T in ORFV is similar to the recently discovered mutation of the host gene APOBEC3A/B driving monkeypox virus (46), but there is little change in the expression level of APOBEC family genes after ORFV infects the host. The C>T conversion may be due to the fact that cytosine in the DNA double strand is often methylated and then spontaneously deaminated to thymine, while the T>C conversion is also seen in varicella vaccine (47, 48) and may be related to virulence. Synonymous SNPs hold specific biological implications in virus evolution, while non-synonymous SNPs leading to amino acid changes can directly impact viral virulence (49). Notably, ORF018 encodes the catalytic subunit of poly-A polymerase, which plays a critical role in gene expression control (50). The variation of its amino acid may alter its activity, consequently affecting viral gene expression and proliferation. ORF019 and ORF020 share homology with Vaccinia viruses E2L and E3L, respectively, which are involved in IFN production and facilitation of viral particle transport (51, 52). Variations in ORF019 and ORF020 may affect the ability of viral particles to inhibit IFN. Besides, ORF025 encodes DNA polymerase (53), and its non-synonymous mutation in this gene may cause changes in the replication and proliferation processes of strains with different virulence, potentially contributing to differences observed in virus growth curves. However, it remains unclear whether these SNP events are specific to the FX0910 and aFX0910 strains and whether they depend on host cell-specific binding factors.

aFX0910 proliferates at late stages of infection by modulating the host innate immune response. Cluster analysis of transcript data revealed two distinct branches at 24 hpi, with samples infected with the aFX0910 strain forming a separate cluster at 48 hpi. This suggests that the extent of transcriptome changes in the cell samples is related to the time of infection, emphasizing that the largest transcriptome alterations in response to different virulent strains occur at 48 hpi. Comparative analysis of DEGs in GO and KEGG analyses indicates that ORFV strains with distinct virulence elicit diverse host response mechanisms. FX0910 elicits a strong innate immune response, while aFX0910 upregulates the related chemokines CXCL5, CXCL8, and TGF-β2 to regulate the innate immune response. These findings provide a theoretical reference for utilizing aFX0910 as an attenuated vaccine.

Among these DEGs, immune-related genes were also changed to varying degrees. FX0910 infection induced the expression of a multitude of interferon-stimulated gene (ISG) and interferon regulatory factor (IRF) family genes, activating the host’s innate immune response against viral infection. This response pattern is consistent with other epithelial cell’s response to viral infection (36, 37, 54), possibly mediated by recruitment of upstream transcription factors. In contrast, only a limited number of differentially expressed genes associated with innate immunity were identified in host cells infected with aFX0910. This phenomenon may be attributed to the progressive adaptation of the aFX0910 strain to the host cell’s antiviral response, which was acquired through consecutive passaging (55, 56). Moreover, promoter regions of genes associated with antiviral infection and significantly upregulated in host cells showed enrichment of transcription factors such as STAT1 and STAT2, emphasizing their crucial role in the antiviral defense mechanism. Conversely, aFX0910-infected host cells exhibited only a few DEGs associated with innate immunity, likely indicating the gradual adaptation of aFX0910 strains to the host cell’s antiviral response following successive passages (55, 56).

While the expression patterns of immune genes induced by ORFV infection are generally similar across different species (39, 57, 58), our study revealed distinct host transcriptomic responses to wild-type and attenuated ORFVs. Additionally, the occurrence of SNP events differed in viral transcripts. The licensed ORFV vaccines currently in use mainly consist of live and attenuated live vaccines (59). However, the method of cell passage to attenuate is still expensive and time-consuming, and there is a risk of mutation and reversion to virulent strain. Understanding the changes in host immune responses and viral expression patterns in attenuated strains can enhance our knowledge of ORFV adaptability during the cell passage process and the interplay between ORFV and host cells. These findings provide a theoretical reference for elucidating the mechanism of action of ORFV-attenuated vaccines and for the development of novel vaccines.

## Data Availability

The RNA-seq data of this study have been deposited in NCBI SRA database (PRJNA1021021).

## SUPPLEMENTAL MATERIAL

Supplemental figures msphere.00398-23-s0001.docx Figure S1 to S3.

Supplemental tables msphere.00398-23-s0002.xlsx Tables S1 to S3.

ASM does not own the copyrights to Supplemental Material that may be linked to, or accessed through, an article. The authors have granted ASM a non-exclusive, world-wide license to publish the Supplemental Material files. Please contact the corresponding author directly for reuse.
