# Supplementary material for: Dual analysis of wild-type and attenuated Orf virus and host cell transcriptomes revealed novel virus-host cell interactions
Source: mSphere. 2023 Nov 20;8(6):e00398-23. doi: 10.1128/msphere.00398-23 (PMC10732022; doi:10.1128/msphere.00398-23)
Supplement: Supplemental figures — Figure S1 to S3. [file msphere.00398-23-s0001.docx]

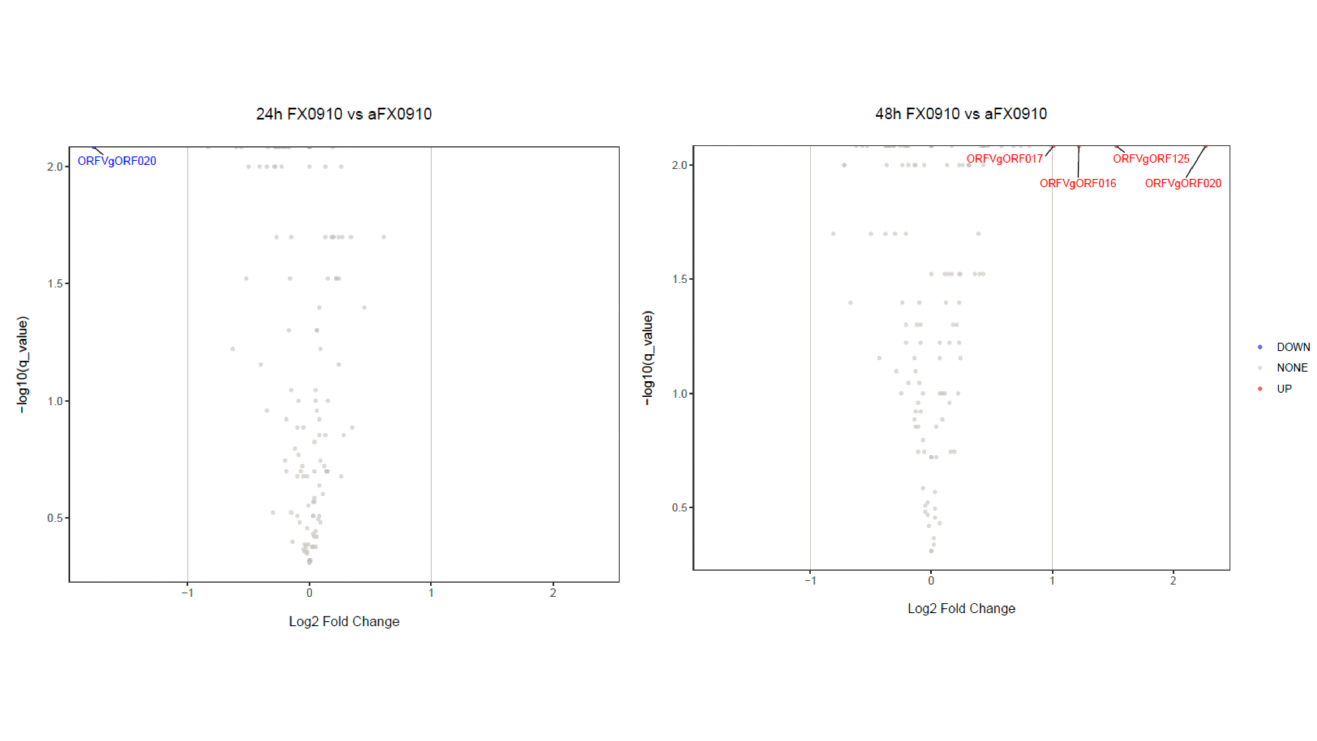


Figure S1. Differential expression profile of virus genes (|log2 fold change| > 1). The left plot shows the viral gene differentially expressed by FX0910 and aFX0910 24h after virus infection; The right plot shows the viral gene differentially expressed between FX0910 and aFX0910 after 48h of virus infection. Among them, red point indicates up-regulated genes, and blue point indicates down-regulated genes.


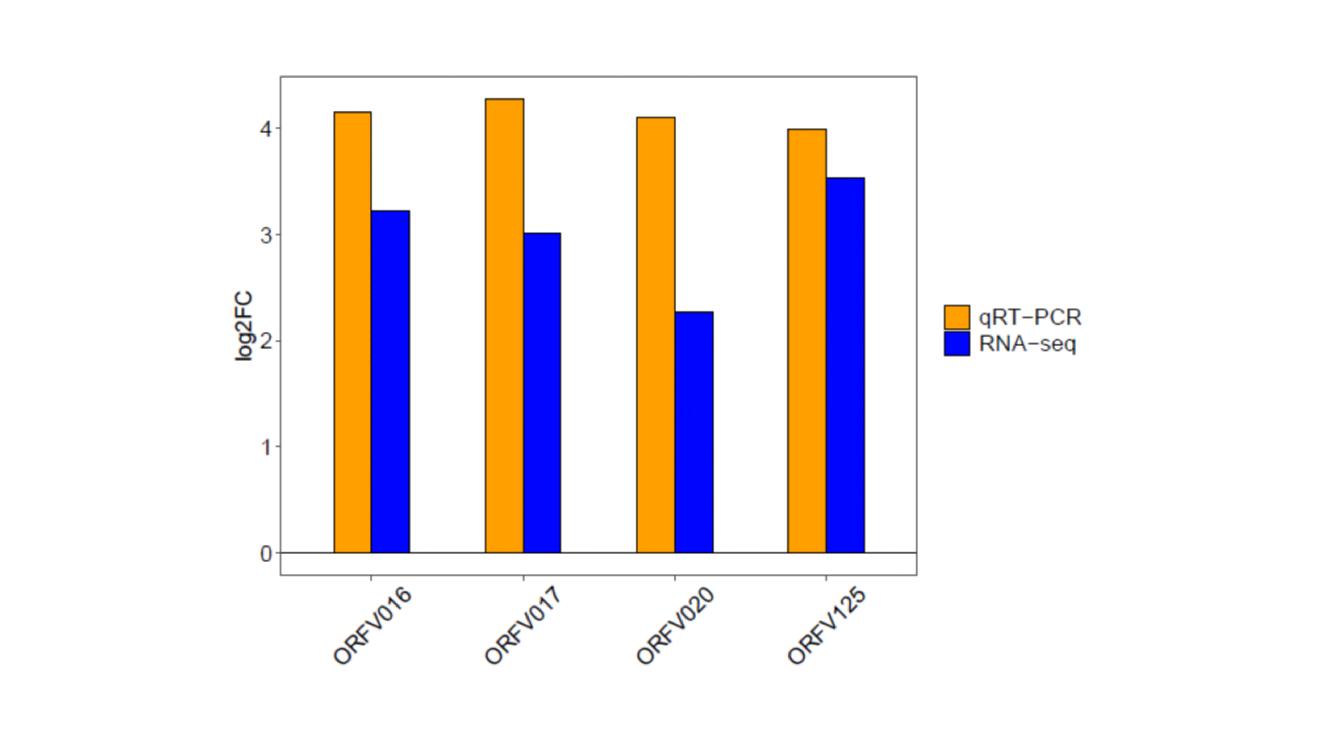
Figure S2. At 48 hours after FX0910 infection, real-time PCR validation results of differential expression of virus genes. The vertical axis in the figure is the up-regulation and down-regulation of gene expression, in which positive values represent up-regulated expression and negative values represent down-regulated expression.


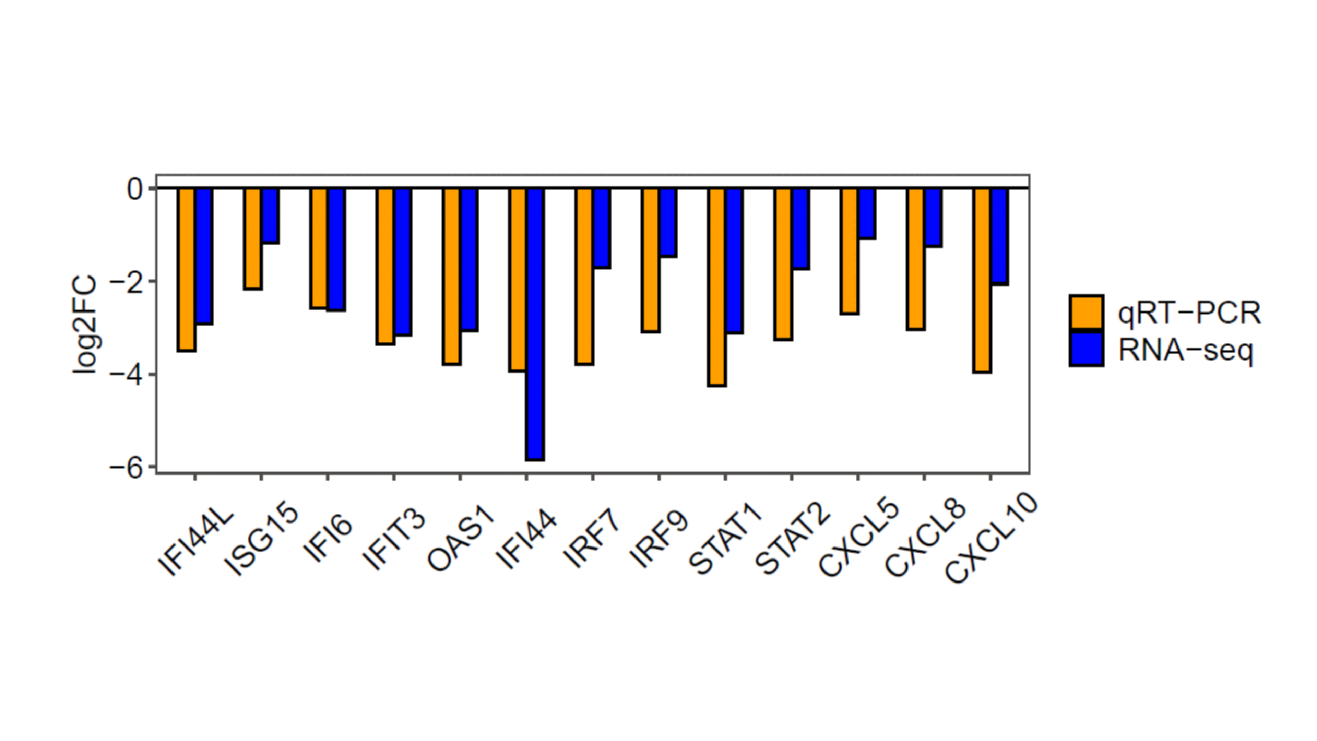


Figure S3. At 48 hours post-infection, real-time PCR validation results of differential expression of host genes (aFX0910 infection vs FX0910 infection). The vertical axis in the figure is the up-regulation and down-regulation of gene expression, in which positive values represent up-regulated expression and negative values represent down-regulated expression.
